# Prenatal inhibition of the kynurenine pathway leads to structural changes in the hippocampus of adult rat offspring

**DOI:** 10.1111/ejn.12535

**Published:** 2014-03-19

**Authors:** Omari S Khalil, Mazura Pisar, Caroline M Forrest, Maria C J Vincenten, L Gail Darlington, Trevor W Stone

**Affiliations:** 1 Institute of Neuroscience and Psychology, West Medical Building, University of GlasgowGlasgow, G12 8QQ, UK; 2 Epsom General HospitalEpsom, Surrey, UK

**Keywords:** doublecortin, GABA transport, glutamate transport, kynurenic acid, neurodevelopment, sonic hedgehog

## Abstract

Glutamate receptors for *N*-methyl-d-aspartate (NMDA) are involved in early brain development. The kynurenine pathway of tryptophan metabolism includes the NMDA receptor agonist quinolinic acid and the antagonist kynurenic acid. We now report that prenatal inhibition of the pathway in rats with 3,4-dimethoxy-*N*-[4-(3-nitrophenyl)thiazol-2-yl]benzenesulphonamide (Ro61-8048) produces marked changes in hippocampal neuron morphology, spine density and the immunocytochemical localisation of developmental proteins in the offspring at postnatal day 60. Golgi–Cox silver staining revealed decreased overall numbers and lengths of CA1 basal dendrites and secondary basal dendrites, together with fewer basal dendritic spines and less overall dendritic complexity in the basal arbour. Fewer dendrites and less complexity were also noted in the dentate gyrus granule cells. More neurons containing the nuclear marker NeuN and the developmental protein sonic hedgehog were detected in the CA1 region and dentate gyrus. Staining for doublecortin revealed fewer newly generated granule cells bearing extended dendritic processes. The number of neuron terminals staining for vesicular glutamate transporter (VGLUT)-1 and VGLUT-2 was increased by Ro61-8048, with no change in expression of vesicular GABA transporter or its co-localisation with vesicle-associated membrane protein-1. These data support the view that constitutive kynurenine metabolism normally plays a role in early embryonic brain development, and that interfering with it has profound consequences for neuronal structure and morphology, lasting into adulthood.

## Introduction

There is increasing evidence to suggest that the earliest stages of brain development *in utero* may be susceptible to modification by environmental factors, including diet, stress, and infection, which affect the rate of brain development overall or the relative development of different brain regions, leading to the appearance of disorders such as schizophrenia in postnatal life (Meyer & Feldon, 2010; Brown, 2011). However, it is not clear, at the molecular level, how such prenatal influences could impact on cerebral development.

One biochemical pathway that is ideally situated to link the environment and the brain is the kynurenine pathway, the major route of tryptophan metabolism in mammals (Stone & Darlington, 2002, 2013). The oxidation of tryptophan to kynurenine leads to the generation of two compounds with known activity at *N*-methyl-d-aspartate (NMDA) receptors. These are quinolinic acid, an agonist selective for NMDA receptors (Stone & Perkins, 1981; Stone, 2001), and kynurenic acid, which is able to antagonise glutamate at all of its ionotropic receptors, although with the greatest potency in blocking the Gly2 site of the NMDA receptor (Perkins & Stone, 1982; Stone *et al*., 2013). Kynurenic acid may also block nicotinic cholinoceptors (Hilmas *et al*., 2001), although others have failed to confirm this observation (Mok *et al*.,^2009^; Dobelis *et al*., 2012).

It is well established that, in the course of early brain development, NMDA receptors are involved in the formation of neurites and axon branches, the guidance of growth cones towards their targets, and the eventual establishment of synaptic contacts (Rajan & Cline, 1998; Colonnese *et al*., 2005; Alvarez *et al*., 2007; Ultanir *et al*., 2007). These and other aspects of neuronal and synaptic development ultimately determine synaptic function and plasticity in the offspring (Iwasato *et al*., 2000; Ramoa *et al*., 2001). It is therefore possible that the kynurenine pathway, by modulating the activation and blockade of NMDA receptors, is involved in these early stages of brain development in the embryo. As the pathway is influenced by infection, which leads to the induction of the first enzyme of the pathway (indoleamine-2,3-dioxygenase) via interferon-γ, and stress, which activates tryptophan-2,3-dioxygenase via glucocorticoid release, levels of the neuroactive kynurenine metabolites are likely to change to a degree that would affect NMDA receptor function, and thus cerebral development. Indeed, we have shown previously that inhibiting the kynurenine pathway *in utero* results in changes in protein expression (including for NMDA receptor subunits), neuronal excitability or synaptic plasticity as early as 5 h after treatment, with significant changes persisting to weaning at postnatal day (P) 21 and adulthood at P60 (Forrest *et al*., 2013a,b).

In the present study, we treated pregnant rats in late gestation with 3,4-dimethoxy-*N*-[4-(3-nitrophenyl)thiazol-2-yl]benzenesulphonamide (Ro61-8048), an inhibitor of kynurenine-3-monoxygenase (KMO), which has been shown to increase the concentration of kynurenic acid within the central nervous system (CNS) (Röver *et al*., 1997; Clark *et al*., 2005; Forrest *et al*., 2013a). The offspring were then allowed to litter normally and develop until P60, a time at which behavioural changes have been reported after prenatal infection or the administration of infection-mimetic agents (Fatemi *et al*., 2005; Zuckerman & Weiner, 2005; Meyer & Feldon, 2010). Although many proteins were unchanged by Ro61-8048 treatment at P21 and P60, there were significant changes in NMDA receptor subunits and the cell generation and maturation proteins doublecortin (DCX) and sonic hedgehog (Shh). Here, we sought to determine whether the modified expression of these proteins is associated with structural changes at the cellular level that might account for the alterations in synaptic function, as well as any associated effects on neuronal dendritic field structure and spine density. We also sought to visualise and localise the changes in Shh and DCX expression. The work reported is focused on the hippocampus, as our previous work has shown functional changes in synaptic transmission and plasticity in this region (Forrest *et al*., 2013a), and it was intended to produce further evidence on the nature and magnitude of cellular changes that might explain, or contribute to, those functional modifications. The hippocampus is believed to be important in a range of cognitive processes (Sweatt, 2004), and could be a pharmacological target in psychiatric disorders such as schizophrenia (Harrison, 2004). Overall, the results indicate that prenatal inhibition of the kynurenine pathway produces marked effects on neuronal structure and development, suggesting that the pathway is constitutively active in the embryo and contributes to the regulation of early brain development.

## Materials and methods

The study was approved by the Glasgow University College of Medical, Veterinary and Life Sciences Ethics Committee. The project was licensed by, and carried out according to the conditions of, the UK Home Office under the Animals (Scientific Procedures) act 1986, administered and monitored by the Home Office and in accordance with the Council Directive 2010/63EU of the European Parliament and the Council of 22 September 2010 on the protection of animals used for scientific purposes. After mating, female Wistar rats were examined daily for the appearance of a vaginal plug, at which time three pregnant rats were taken for each treatment group. These were then separated from the males and housed alone with free access to food and water.

Inhibition of the kynurenine pathway was achieved with Ro61-8048 (Röver *et al*., 1997). This compound inhibits KMO, the enzyme responsible for converting kynurenine to 3-hydroxykynurenine (Stone & Darlington, 2002, 2013). This inhibition results in increased availability of kynurenine for transamination to kynurenic acid, and, in our previous work, has increased kynurenic acid concentrations in the pregnant dam and the embryo brains by 10–100-fold (Forrest *et al*., 2013a), which is similar to the increase obtained in earlier studies using KMO inhibitors in adult, non-pregnant animals (Chiarugi *et al*., 1995; Speciale *et al*., 1996; Röver *et al*., 1997; Clark *et al*., 2005). The dose of Ro61-8048 used was 100 mg/kg, injected intraperitoneally, which we have found to be capable of repeated administration with no ill-effects on the animals treated (Clark *et al*., 2005; Rodgers *et al*., 2009). Control rats were given the vehicle (0.9% sodium chloride solution adjusted to pH 7). The compound was administered on three occasions in late gestation – embryonic day (E) 14, E16, and E18 – to maximize the period of development during which the activity of the kynurenine pathway is affected. Gestation, parturition and weaning were then allowed to proceed normally, with offspring being killed at P60 for the removal of brains. Litter size tended to be lower in the treated rats, with a mean of 7.4 ± 1.2 (*n* *=* 9) pups per dam, than in controls (10.2 ± 1.1 pups per dam, *n* *=* 9), but the difference was not significant (two-tailed *t*-test, *P* = 0.1). For the work described below, groups of two or three rats from three or four litters were used per treatment with vehicle or Ro61-8048, giving a total of 7–12 offspring in each group.

### Golgi staining and Sholl analysis

At P60, rats were killed by an intraperitoneal injection of sodium pentobarbitone, and the brains were removed. Sample preparation and Golgi silver staining were performed with the FD Rapid GolgiStain Kit (FD Neurotechnologies, Columbia, MD, USA), according to the manufacturer's instructions. Brains were immersed in the impregnation solution for 2–3 weeks in the dark at room temperature, the solution being refreshed after the initial 24 h of immersion. The tissue was transferred into solution C and stored at 4 °C for at least 48 h, the solution again being replenished after the first 24 h. The brains were then cut into 200-μm coronal slices with a Leica VT1200 series Vibratome (Leica Microsystems, Milton Keynes, UK). Each section was mounted on a gelatine-coated slide and allowed to dry at room temperature, shielded from light. Once dried, the sections were rinsed twice for 2 min, and placed into a mixture of solutions D and E of the GolgiStain Kit for 10 min. Finally, the sections were rinsed twice for 4 min, and then progressively dehydrated in 50, 75 and 95% ethanol for 4 min each, followed by 100% ethanol for four 4-min periods. Sections were cleared in Histoclear three times for 4 min each, and covered with a coverslip by the use of Histomount (ThermoFisher Scientific, Loughborough, UK). The chemical constitution of the commercial solutions has not been disclosed by the manufacturers.

### Dendritic morphology

In the CA1 stratum pyramidale, an average of six to eight pyramidal neurons were examined in each of three or four hippocampal sections from each rat, with a Nikon Eclipse E400 microscope, by an investigator blind to the treatment of the rat. Neurons were inspected with a ×40 objective lens, and a copy of each neuron was drawn with a camera lucida onto a two-dimensional plane measuring 420 × 297 mm. The criteria for neuron selection for analysis were that neurons were clearly delineated along their entire length and breadth by a consistent and clearly visible level of silver staining, and that each neuron was sufficiently distinct from neighbouring impregnated neurons to avoid contamination of the measurement process by elements of nearby cells. To ensure that only complete cells were reconstructed, the selected cells were located close to the centre of the section, and superficial cells, with significant processes cut at the surface of the section, were excluded from analysis.

Morphological data were primarily analysed with an unpaired *t*-test to compare differences in soma size, dendrite number and dendrite length between control and Ro61-8048-treated groups of rats. Apical and basal dendrites were examined separately for CA1 pyramidal neurons, whereas only apical dendrites were quantified in the dentate gyrus granule cells, where basal dendrites are few in number and rudimentary. For analysis of dendritic branching complexity (Sholl, 1953), a two-way anova was performed, followed by Tukey's *post hoc* analysis to assess the contributions of drug treatment, sample number, and distance from the soma, with a *P*-value of 0.05 being taken as the criterion for significance.

### Spine density

Spine densities on the dendrites of CA1 pyramidal neurons were observed under a Nikon Eclipse E400 microscope with a ×100 objective lens with oil immersion by an investigator blind to the treatment group. Spines were traced and counted on a two-dimensional plane, with identification of thick and thin spines based on the criteria of Harris *et al*. (1992), where spines were classified as having a mushroom profile if the diameter of the head was greater than the diameter of the neck, and as thin if the diameter of the head and neck were similar and the length was greater than the neck diameter. Three apical or basal dendritic segments per cell and a total of four cells per rat from 11 or 12 rats per treatment group were used for analysis. The data were analysed with an unpaired *t-*test to compare differences between control and Ro61-8048-treated rats in the overall density of spines per 10-μm length of dendrite and the numbers of mushroom spines and thin spines. A *P*-value of 0.05 was taken as the working criterion for significance.

### Immunocytochemistry

Animals were deeply anaesthesised with sodium pentobarbitone (Euthatal) and perfused transcardially via the left ventricle with 50 mL of artificial cerebrospinal fluid (115 mm NaCl; 25 mm HCO_3_; 2.2 mm KH_2_PO_4_; 2 mm KCl; 1.2 mm MgSO_4_; 2.5 mm CaCl_2_; 10 mm d-glucose; gassed with 5% CO_2_ in O_2_) followed by approximately 100 mL of 4% paraformaldehyde in 0.2 m phosphate-buffered saline (PBS) (pH 7.2). Immediately following perfusion, the brains were removed and fixed in the buffered paraformaldehyde solution for a further 4 h at 4 °C. After thorough rinsing in 0.1 m PBS, the brains were protected in a 30% sucrose solution at 4 °C until saturated. Sections (60 μm) were cut coronally (approximately −2.5 to −3.5 mm relative to bregma) with a vibratome (Leica VT1200; Leica), and collected serially in 0.1 m PBS. The sections were incubated with 50% ethanol for 30 min before being washed three times with 0.3 m PBS and stored in glycerol at −20 °C.

Immunocytochemistry for vesicle-associated membrane protein-1 (VAMP-1), vesicular glutamate transporter (VGLUT)-1/2 and vesicular GABA transporter (VGAT) was carried out with the following primary antibodies: anti-VAMP-1/synaptobrevin (goat polyclonal, AF4828, 1 : 250 dilution) (R&D Systems, Abingdon, UK), anti-VGLUT-1 (rabbit polyclonal, 1 : 500 dilution), anti-VGLUT-2 (rabbit polyclonal, 1 : 500), and anti-VGAT (mouse monoclonal, 1 : 1000 dilution) (Synaptic Systems, Goettingen, Germany).

Three to four coronal hippocampal sections per rat were examined with a BioRad Radiance 2100 confocal laser scanning system equipped with lasers – argon (488 nm), green helium neon (543 nm), and red diode (637 nm) – in conjunction with lasersharp 2000 software. Pyramidal cell bodies in the CA1 region were scanned with a ×60 oil immersion objective lens (numerical aperture, 1.4; image size, 1024 × 1024) with a zoom factor of 3 (yielding a pixel size of 0.07 μm). Each field consisted of a stack of 30 optical sections with an increment of 0.3-μm z-separation. In an attempt to count the individual punctate staining for analysis, the contrast of each colour channel was manually adjusted within the maximum range to minimise the fusion of puncta (Fattorini *et al*., 2009). Individual punctate staining was manually counted in every third subfield in an area measuring 50 × 50 μm from the 1024 × 1024-pixel images. Each channel for VAMP-1, VGLUT-1/2 and VGAT was analysed separately to identify and manually count immunopositive puncta. Co-localisation of VAMP-1 with VGLUT-1/2 or VGAT was analysed by merging the two relevant channels and manually counting the number of co-localised puncta. Punctate staining was considered to be co-localised when the overlap was complete or occupied most of the punctate area (Bragina *et al*., 2010). The counting of punctate staining was performed with imagej software.

For analyses of hippocampal neurogenesis, serial free-floating sections were first washed in 0.3 m PBS before incubation with primary antibodies in PBS containing 0.1% Triton-X100 (pH 7.4) for 72 h at 4 °C with continuous agitation. The primary antibodies used were monoclonal anti-NeuN 1 : 500 (for neuronal nuclei; MAB377; Millipore, Watford, UK), polyclonal anti-DCX 1 : 250 (sc-8066; Santa Cruz via Insight Biotechnology, Wembley, UK), and polyclonal anti-Shh 1 : 100 (sc-9024; Santa Cruz). Following primary antibody treatment, sections were incubated overnight at 4 °C in species-specific Alexa Fluor-tagged secondary antibodies (Alexa 488; Alexa 647; Molecular Probes, Life Technologies, Paisley, UK) and rhodamine-conjugated secondary antibody (Jackson Immunoresearch Laboratories, Stratech, Newmarket, UK). Rinses were performed between all steps with 0.3 m PBS before or after the primary and secondary antibody incubation. Sections were mounted and coverslipped with the aqueous mounting anti-fade medium H-1000:Vectashield (Vector Laboratories, Peterborough, UK) before storage at −20 °C. Granule cells of the dentate gyrus and pyramidal cell bodies in CA1 and CA3 were scanned with most parameters similar to those outlined above, but with a ×40 oil immersion objective lens. Each field consisted of a stack of 20 optical sections with an increment of 1-μm z-separation. Immunopositive staining was manually counted in each subfield in an area measuring 50 × 50 μm from the 1024 × 1024-pixel images. Each channel for Shh, NeuN and DCX was analysed separately to identify and manually count immunopositive staining with imagej software. The overall dendrite length of DCX-labelled granule cells in the dentate gyrus was measured with the imagej plug-in neuronj. A *P*-value of 0.05 was taken as the working criterion for significance.

### Data analysis

Statistical comparisons were made by the use of instat software, between pups born to mothers injected with vehicle and pups born to those treated with Ro61-8048. This protocol allowed the use of an unpaired, two-tailed *t*-test to examine differences between the two groups for each treatment. Data were initially analysed to compare results between male and female offspring, but no significant differences were noted in the various parameters measured, so the data were combined and analysed together. For the analysis of dendritic complexity, anova was used, followed by Tukey's multiple comparison test. A *P *< 0.05 was used as the criterion for significance.

## Results

### CA1 pyramidal neurons

Measurement of the maximum diameters of neuronal somata in CA1 did not reveal any significant changes in soma size between the rats exposed *in utero* to Ro61-8048 and the control rats exposed only to vehicle. Somatic size in the controls was 15.79 ± 0.51 μm (*n* *=* 12 rats), and that in the drug-treated group was 14.75 ± 0.52 μm (two-tailed *t*-test, *P* = 0.274; *n* = 12 rats).

### Dendritic measurements

**Figure 1:**
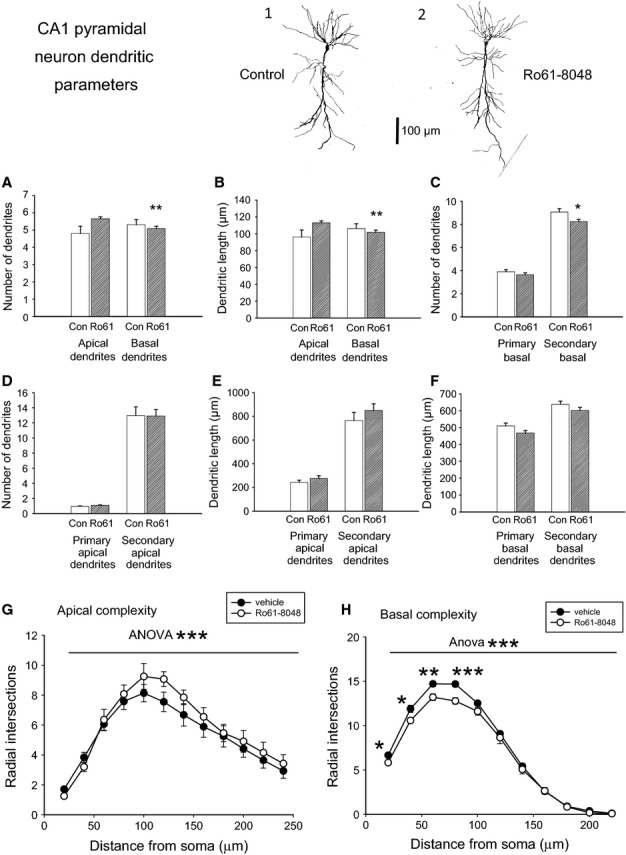
Morphological analysis of silver-stained neurons in the hippocampal CA1 region. Tracings are shown of typical pyramidal neurons from a rat treated with vehicle (1) and a rat treated with Ro61-8048 (2). Scale bar: 100 μm. (A) The total number of dendrites in the apical and basal dendritic trees. (B) The total length of the dendrites in the apical and basal dendritic trees. (C) The number of primary and secondary dendrites in the basal tree, with significant differences between control and vehicle-treated tissue (D) This indicates the absence of any difference in the apical dendrites. (E and F) A similar analysis of dendritic length. Data from control, vehicle-treated rats are shown as open bars, and data from Ro61-8048-treated rats are shown as shaded bars. (G and H) Analysis of dendritic complexity is illustrated for the apical (G) and basal (H) dendrites. *P < 0.05; **P < 0.01; ***P < 0.001.

Several aspects of the dendritic tree were analysed. Counting each branch point generated a value for the number of dendritic branches in the tree. This measurement was made separately for dendrites in the apical and basal regions of CA1 pyramidal cells, with a significant loss being observed in the number of basal but not of apical dendrites (Fig.1A). A small but highly significant decrease was also noted in the length of the basal dendritic tree (Fig.1B).

When the data were analysed in terms of the relative contributions of the primary and secondary dendrites in each dendritic region, a significant difference was found between the groups, with significantly fewer secondary dendrites (two-tailed *t*-test, *P* = 0.047, *n* = 12) being observed in the basal dendritic regions of hippocampal sections from rats treated with Ro61-8048 (Fig.1C). In contrast, there was no difference in the number of secondary dendrites in the apical dendritic regions (Fig.1D) (with only a single primary apical dendrite by definition).

Despite the decreased overall length of basal dendrites, there were no differences at the level of the primary or secondary apical tree (Fig.1E) or basal tree (Fig.1F).

Finally, a comparison was made of the dendritic branching complexity between the control and the Ro61-8048-treated rats. The Sholl assessment of this parameter requires counting the number of intersections of dendrites with each ring of a series of 20-μm-spaced concentric circles on the analysis plan. The counts were compared by use of a multifactorial analysis of variance employing a 2 × 12 × 24 anova based on drug treatment, distances of intersection for the Sholl analysis, and number of samples included in the analysis. There were significant effects of Ro61-8048 on treatment group (*F*_1,242_ = 6.90; *P* < 0.01), intersection distance (*F*_11,242_ = 49.46; *P* < 0.001) and sample (*F*_22,242_ = 8.33; *P* < 0.001) for the apical dendritic tree, with no significant interaction between drug and distance (*F*_11,242_ = 0.93; *P* = 0.51) (Fig.1G) and no significant differences between individual data points. Analysis of the dendritic branching complexity of the basal dendrites also revealed significant effects of treatment (*F*_1,242_ = 30.18; *P* < 0.001), distance (*F*_11,242_ = 776.46; *P* < 0.001), and sample (*F*_22,242_ = 3.33; *P* < 0.001), with a significant interaction between treatment and distance (*F*_11,242_ = 2.75; *P* < 0.01). Tukey's *post hoc* analysis indicated a significant decrease in basal dendritic branching complexity for hippocampi exposed to Ro61-8048 as compared with the control group at 20 μm (two-tailed *t*-tests, *P* < 0.05), 40 μm (*P* < 0.05), 60 μm (*P* < 0.01) and 80 μm (*P* < 0.001) from the soma (Fig.1H).

### Spine density and morphology

**Figure 2:**
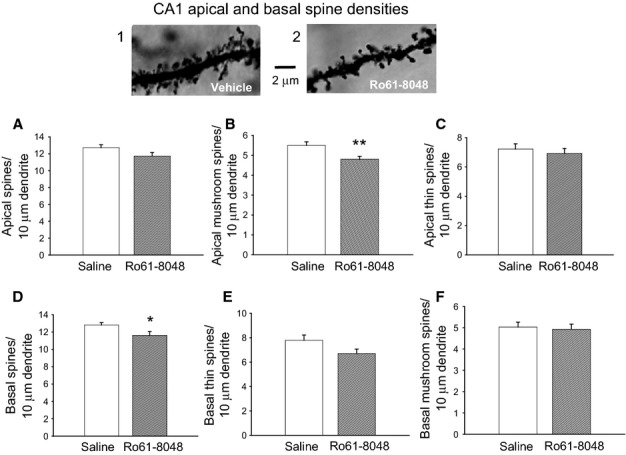
Effects of prenatal Ro61-8048 treatment on adult hippocampal CA1 dendritic spines. The photomicrographs show spines on an apical dendrite from a rat treated with vehicle (1) and a rat treated with Ro61-8048 (2). (A) Overall spine density. (B and C) Separately quantified densities of mushroom spines (B) and thin spines (C) in the CA1 apical dendrites. (D–F) Corresponding data for total spines (D), thin spines (E) and mushroom spines (F) on basal dendrites. Mushroom and thin spines were counted in successive 10-μm lengths of pyramidal neurons. *P < 0.05; **P < 0.01.

In the apical dendritic tree, the total spine density was no different in the Ro61-8048-exposed tissue (Fig.2A), although separate analysis of the thin and thick (mushroom-shaped) spines indicated a significant reduction in the number of the latter (two-tailed *t*-test, *P* = 0.007) (Fig.2B) with no significant difference in the population of thin spines (Fig.2C; two-tailed *t*-test, *P* = 0.54). The density of spines on the basal dendrites of CA1 neurons was significantly lower in tissue from rats exposed to Ro61-8048 *in utero* than in control tissue (two-tailed *t*-test, *P* = 0.036) (Fig.2D), but with no differential loss of thin spines (two-tailed *t*-test, *P* = 0.07) (Fig.2E) or thick mushroom spines (*P* = 0.75; Fig.2F).

### Immunocytochemistry

As the initial postulate of this work was that altered activity along the kynurenine pathway would affect NMDA receptor activation and thus brain development, a primary objective was to ascertain whether any changes were produced in glutamatergic neuron function. To this end, the number of synaptic boutons containing VGLUT-1 or VGLUT-2 and their co-localisation with the synaptic terminal marker VAMP-1 (synaptobrevin) were determined. Punctate staining was counted manually within a box size of 50 × 50 μm, and co-localisation of VAMP-1 was assessed with VGAT (yellow) and VGLUT-1/2 (light blue).

**Figure 3:**
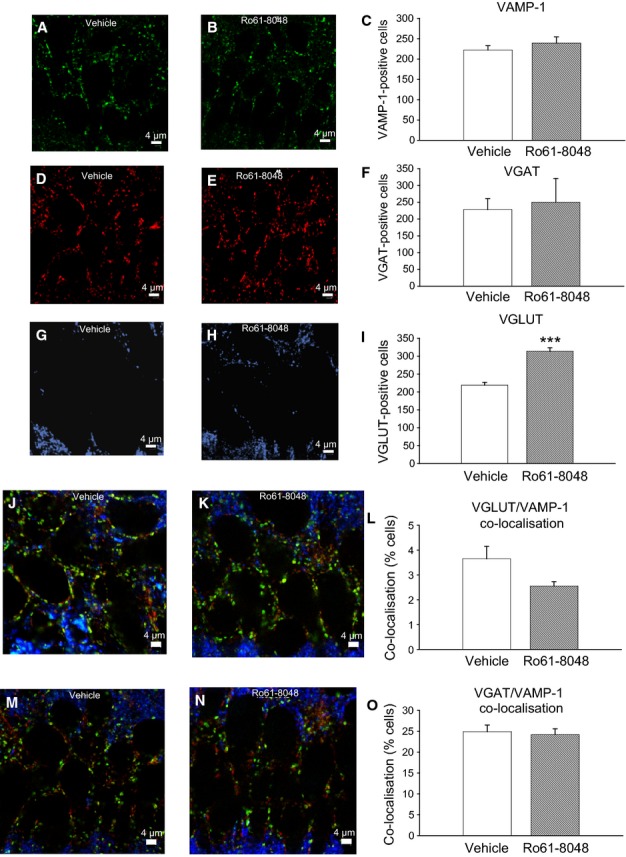
Glutamate and GABA transporters after prenatal Ro61-8048 treatment. (A–C) The number of nerve terminals immunoreactive for VAMP-1 is illustrated by the sample micrographs (A and B) and the quantitative bar chart (C). (D–F) No significant difference was noted between vehicle-treated and Ro61-8048-treated tissue for VAMP-1 or VGAT. (G–I) There was a significantly larger number of terminals positive for VGLUT-1/2. (J–O) Sample combined images of staining for VGLUT and VAMP-1 are shown in J and K, and combined images for VGAT and VAMP-1 immunoreactivity in M–O but with no significant differences detected (L,O). Scale bars: 4 μm. ***P < 0.001.

There was no overall change in the number of cells immunopositive for VAMP-1 (Fig.3A–C) or VGAT (Fig.3D–F). Staining for VGLUT revealed a widespread distribution of labelled terminals throughout CA1. As summarized in Fig.3G–I, there was a substantial increase in the number of terminals staining for these transporters in rats exposed to Ro61-8048 (two-tailed *t*-test, *P* = 0.0003, *n* = 8 per group). An assessment of the co-localisation of VGLUT or VGAT and VAMP-1 indicated no change in the fraction of cells staining for both proteins (Fig.3J–L and M–O), although the number of cells identified with VGLUT and VAMP-1 was small.

**Figure 4:**
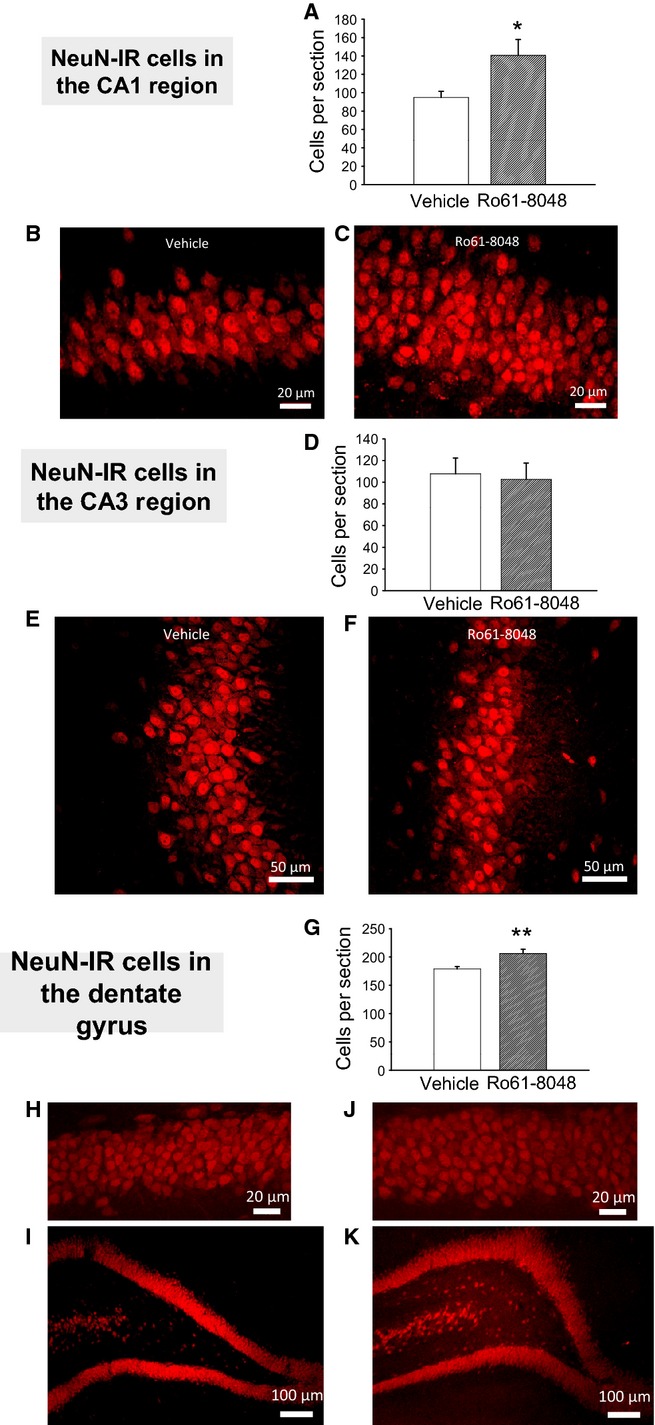
Immunocytochemical examination of NeuN in the adult hippocampus after prenatal Ro61-8048 treatment. (A–C) Cell counts of CA1 NeuN-immunoreactive (NeuN-IR) pyramidal neurons (A), with sample photomicrographs from vehicle-treated (B) and Ro61-8048-treated (C) rats. (D–F) Comparable results for CA3 pyramidal cells. (G–K) The number of stained granule cells in the dentate gyrus (G), with photomicrographs at high magnification (H and J) and low magnification (I and K). Scale bars: 20 μm in B, C, H, and J; 50 μm in E and F; 100 μm in I and K. *P < 0.05; **P < 0.01.

NeuN staining was used to provide an indication of the total number of mature neurons in the different hippocampal regions. In the stratum pyramidale of CA1, there was a significant increase in the number of neurons in sections from rats exposed to Ro61-8048 (two-tailed *t*-test, *P* = 0.017, *n* *=* 9 for vehicle, *n* = 7 for Ro61-8048) (Fig.4A–C), although there was no significant difference in the number of neurons in CA3 (*P* = 0.8; Fig.4D–F).

**Figure 5:**
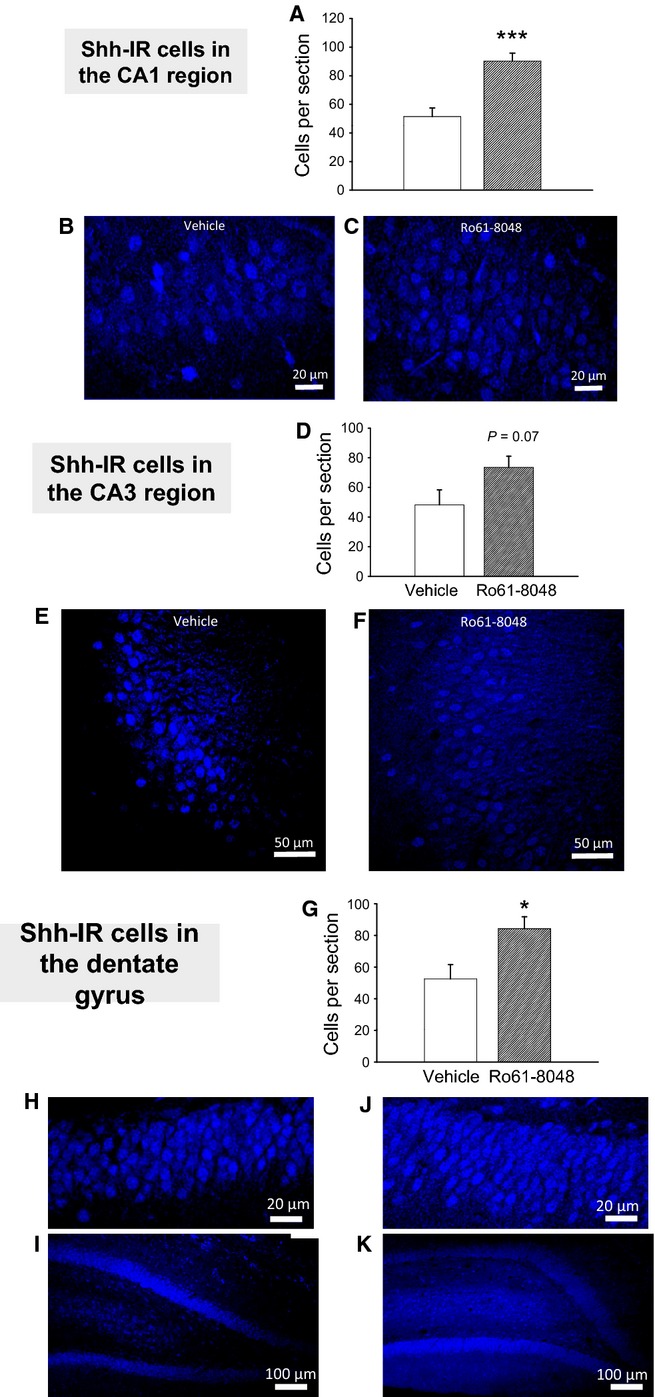
Immunocytochemical examination of Shh immunoreactivity in the adult hippocampus after prenatal Ro61-8048 treatment. (A–C) Cell counts of CA1 Shh-immunoreactive (Shh-IR) pyramidal neurons (A), with sample photomicrographs from vehicle-treated (B) and Ro61-8048-treated (C) rats. (D–F) Comparable results for CA3 pyramidal cells. (G–K) The number of stained granule cells in the dentate gyrus (G) with photomicrographs at high magnification (H and J) and low magnification (I and K). Scale bars: 20 μm in B,C,H,and J; 50 μm in E and F; 100 μm in I and K. *P < 0.05; ***P < 0.001.

Shh is a key protein in the early maturation and morphogenetic organisation of cells. The changes observed in the quantification of Shh immunostaining in CA1 were more pronounced than with any of the other targets examined in this study, reflecting the existence of marked changes in expression of this protein noted in embryonic, P21 and P60 brains (Forrest *et al*., 2013a,b). Very significant changes were observed, with almost double the number of Shh-positive neurons in CA1 (two-tailed *t*-test, *P* = 0.0003, *n* *=* 8 rats per group) (Fig.5A–C) and a 40% increase in mean numbers in CA3, which did not quite achieve significance (two-tailed *t*-test, *P* = 0.07, *n* *=* 9 for vehicle, *n* = 8 for Ro61-8048) (Fig.5D–F).

An examination of CA1 and CA3 revealed almost no staining with DCX, only a few scattered cells being apparent in the pyramidal cell layers; no quantification of these was attempted.

### Dentate gyrus granule neurons

**Figure 6:**
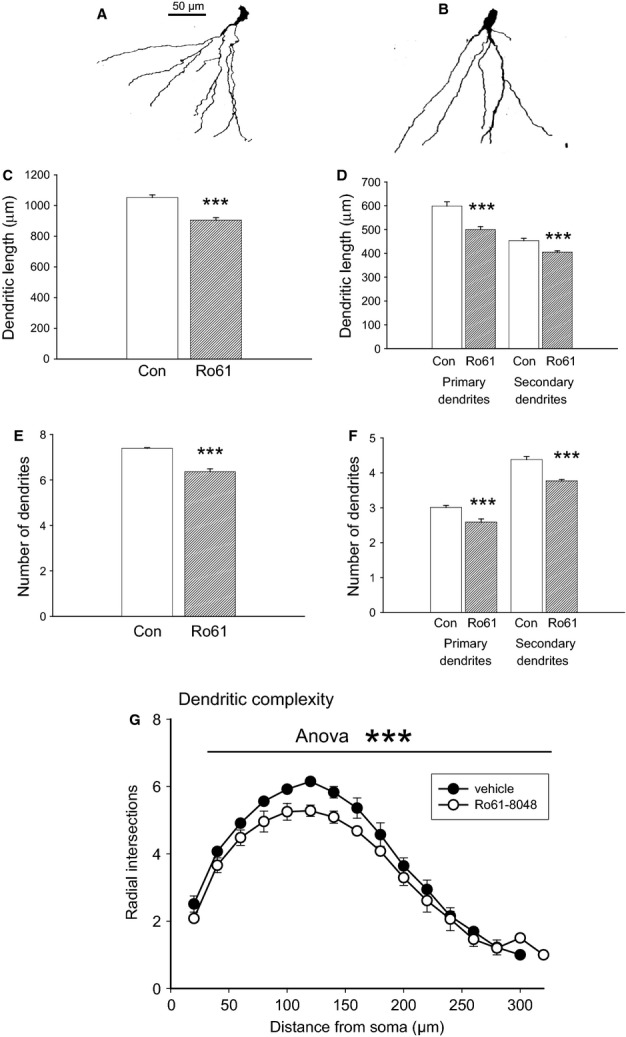
Morphological analysis of silver-stained neurons from the hippocampal dentate gyrus. (A and B) Typical tracings of granule neurons from a rat treated with vehicle (A) and a rat treated with Ro61-8048 (B). Scale bar: 50 μm. (C and D) The total length of dendrites (C) and the length of the primary and secondary dendrites analysed separately (D). (E and F) The total number of dendrites (E) and the separation into primary and secondary groups (F). (G) Analysis of dendritic complexity, revealing a highly significant difference between vehicle-treated and Ro61-8048-treated rats when assessed with two-way anova. ***P < 0.001.

There were no visually obvious differences between the appearance of granule cell somata and the arborisation pattern of their apical dendrites in Ro61-treated rats as compared with control rats (Fig.6A and B). The somata were round or oval in shape, and gave rise to apical dendrites that extended into the molecular layer. There was a lack of basal dendrites in most of the observed neurons, consistent with previous reports that granule cells in normal adult rats possess few, if any, basal dendrites (Arisi & Garcia-Cairasco, 2007).

A comparison of the total dendrite population revealed a significant reduction in the total length of the dendrites (Fig.6C), affecting both primary and secondary dendritic trees analysed separately (Fig.6D). There was also a decrease in the total number of dendrites (Fig.6E), which was again associated with changes in both primary and secondary branches (Fig.6F). For the Sholl assessment of granule cell basal dendritic complexity, intersection counts were compared as described above. There were significant effects of Ro61-8048 on treatment group (*F*_1,234_ = 44.45; *P* < 0.0001), intersection distance (*F*_13,234_ = 213.36; *P* < 0.0001), and sample (*F*_18,234_ = 7.11; *P* < 0.0001), with no significant interaction between drug and distance (*F*_13,234_ = 0.98; *P* = 0.47) (Fig.1G). Overall, the results obtained with this technique strongly support the inference from DCX immunocytochemistry (below) that prenatal treatment with Ro61-8048 resulted in a reduced number and length of dendrites, and reduced complexity of the dendritic system.

### Immunocytochemistry

With the same molecular targets examined in CA1 and CA3, the dentate gyrus was assessed for NeuN and Shh immunoreactivity. There was a significant increase in the number of NeuN-positive granule cells in Ro61-8048-treated rats (two-tailed *t*-test, *P* = 0.005, *n* = 9 for vehicle, *n* = 7 for Ro61-8048) (Fig.4G–K), and approximately double the number of Shh-positive neurons in the dentate gyrus (two-tailed *t*-test, *P* = 0.03, *n* = 9 for vehicle, *n* = 7 for Ro61-8048) (Fig.5G–K). In addition, it was noted that the intercellular matrix of the dentate gyrus of treated rats showed a general staining for Shh that was not present in control specimens (Fig.5I and K). As Shh is a secreted protein (Traiffort *et al*., 2001), this observation may be a result of the increased density of cells reported here secreting normal amounts of the protein. Alternatively, there may be an additional effect of Ro61-8048 treatment on the kinetics of Shh accumulation, such as an increase in the rate or quantity of secretion or a decrease in its removal.

**Figure 7:**
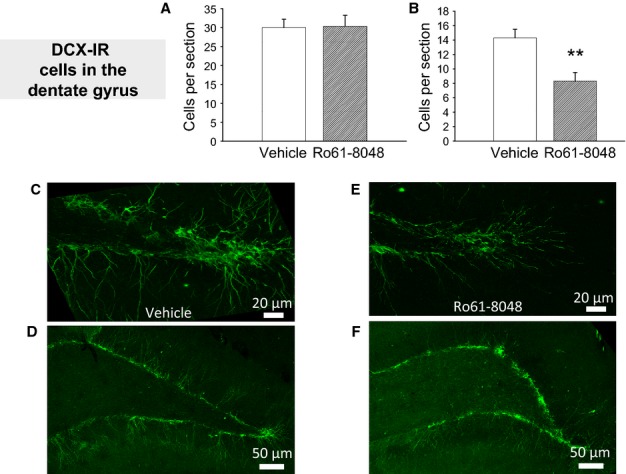
Immunocytochemical examination of DCX immunoreactivity in the adult dentate gyrus after prenatal Ro61-8048 treatment. (A and B) Cell counts of dentate granule DCX-immunoreactive (DCX-IR) neurons and showing short (A) or extensive (B) dendrites. (C–F) Sample photomicrographs from vehicle-treated (C and D) and Ro61-8048-treated (E and F) rats. Scale bars: 20 μm in (C and E); 50 μm in (D and F). **P < 0.01.

Doublecortin is widely used to estimate the number of newly generated and immature neurons in studies of neurogenesis, a phenomenon that is particularly prominent in regions such as the subgranular zone of the hippocampal dentate gyrus. DCX produced clear staining in the dentate gyrus, labelling cells almost exclusively in the granular layer, many of which, especially in control tissue, showed long, clearly stained dendrites. There was no overall effect of Ro61-8048 on the total number of granule cells staining for DCX (Fig.7A), but there was a significant difference in the nature of the dendritic projection (Fig.7B). Dendrites in vehicle-treated rats generally projected at least 50 μm from the cell somata and gave rise to complex networks of fine branches (Fig.7C and D), whereas dendrites in Ro61-8048-treated rats remained relatively simple and unbranched, and often did not extend more than 50 μm from the somata (Fig.7E and F). There were significantly fewer neurons bearing these longer dendrites in tissue from rats exposed to Ro61-8048 (two-tailed *t*-test, *P* = 0.003, *n* *=* 9 for vehicle, *n* = 8 for Ro61-8048) (Fig.7B).

## Discussion

When Ro61-8048 and other KMO inhibitors are administered to adult animals, their major effect is to increase the levels of kynurenic acid in the blood and tissues (Chiarugi *et al*., 1995; Speciale *et al*., 1996; Röver *et al*., 1997; Cozzi *et al*., 1999; Clark *et al*., 2005), including at least 10-fold elevations in brain microdialysates *in vivo* (Urenjak & Obrenovitch, 2000) In the present study, Ro61-8048 was administered to pregnant rats at a dose that has been shown to increase kynurenic acid levels in the blood and brain of the pregnant dam and the brains of the embryos. An increase of 10–100-fold was produced after 5 h *in utero*; the level returned to the control value in the mother after 24 h, but remained little changed in the embryos (Forrest *et al*., 2013a). This indicates that kynurenic acid is produced within the embryo, rather than merely being present as a result of secondary influx from the maternal circulation. This conclusion is supported by the presence of endogenous kynurenic acid in fetal brains at levels higher than those found in the mother (Walker *et al*., 1999). In addition, fetal and placental cells do express indoleamine-2,3-dioxygenase (Ligam *et al*., 2005), so that kynurenic acid can also be generated from either tryptophan or kynurenine from the maternal circulation.

The increase in kynurenic acid level produced would block glutamate receptors in the embryonic brain, especially those sensitive to NMDA (Perkins & Stone, 1982; Stone & Darlington, 2002; Stone *et al*., 2013). Increasing the brain levels of kynurenic acid by the administration of kynurenine and the acidic transport blocker probenecid can increase kynurenic acid levels to a similar degree as that obtained here (Shepard *et al*., 2003), with the ability to reduce quinolinic acid-induced neurotoxicity (Santamaria *et al*., 1996) and neuropathic pain (Pineda-Farias *et al*., 2013), and disrupt sensory gating or prepulse inhibition, as seen in patients with schizophrenia (Shepard *et al*., 2003; Nilsson *et al*., 2006). The endogenous concentration of quinolinic acid is not likely to have been affected, as most previous studies have failed to detect any such change in response to Ro61-8048 (Chiarugi & Moroni, 1999; Clark *et al*., 2005), and quinolinic acid levels do not change even when KMO is completely removed from transgenic mice (Giorgini *et al*., 2013). The objective of this study was to identify structural and neurochemical changes in the adult that might explain the previously described changes in protein expression and synaptic transmission generated by these alterations in kynurenine metabolism (Forrest *et al*., 2013a,b).

### Spine density and neuronal morphology

Neuronal dendrites and their projecting spines receive a majority of the excitatory input to central neurons (Kolb *et al*., 1998), accounting for the close relationship between dendrite and spine numbers, afferent neurotransmission, and learning-related behaviours (Alvarez & Sabatini, 2007), with a probable involvement in a range of neuropsychiatric conditions (Penzes *et al*., 2011).

The density and dendritic distribution of spines is markedly dependent on glutamatergic transmission and the precise composition of the glutamate receptors (Segal & Andersen, 2000). It is particularly intriguing that spine density is affected by the GluN2B subunit (Brigman *et al*., 2010), as Ro61-8048 treatment increased its expression in the embryos after 5 h and in neonates at P21 (Forrest *et al*., 2013a,b). This change could affect the total and relative densities of thick mushroom and thin spines on apical and basal dendrites (Brigman *et al*., 2010). An impact of GluN2B expression on spine formation would help to explain the strong association between GluN2B expression and various aspects of neural plasticity and learning (Mathur *et al*., 2009; Rammes *et al*., 2009; Zhuo, 2009; Fontan-Lozano *et al*., 2011).

Metabotropic glutamate receptor 5 also modifies spine density. Deletion of this receptor results in increased spine density in the neocortex (Chen *et al*., 2012). However, metabotropic glutamate receptor 5 can affect the developmental shift from predominantly GluN2B-containing NMDA receptors to those containing primarily GluN2A, suggesting that NMDA receptors may represent a final common factor in these phenomena. Certainly, the changes in dendritic arborisation are consistent with involvement of the Gly-B site of action of kynurenic acid on the NMDA receptor (Birch *et al*., 1988; Stone, 1993; Stone *et al*., 2013).

The changes in dendritic architecture seen here are likely to have significant effects on neuronal function. The apical and basal dendritic regions have different physiological and pharmacological characteristics, with different plasticity thresholds (Gordon *et al*., 2006; Sajikumar & Korte, 2011) and responses to cholinergic input (Cho *et al*., 2008; Leung & Peloquin, 2010). A differential change in the basal dendrites, the altered location of synapses and the different balance of excitatory and inhibitory synapses that would result may therefore contribute to differences in neuronal excitability and plasticity (Forrest *et al*., 2013a,b). This is especially the case given that minimal changes in the number and calibre of dendritic branches can have a major influence on synaptic input and neural excitability (Ferrante *et al*., 2013).

It is particularly striking that most dendritic parameters were changed by Ro61-8048 in the granule cell population of the dentate gyrus, despite the increased number of neurons implied by increased NeuN-immunopositive cells. This may reflect the presence of more neurons at an early stage of development.

### NeuN

The most fundamental indication of altered cerebral development lies in the number of neurons generated. The hippocampal dentate gyrus is of particular importance in this respect, as it is one of few regions in the adult CNS that shows continuing neurogenesis, primarily in the subgranular zone. The newly formed neurons arise from precursors that, after differentiation, become fully integrated into the pre-existing neuronal circuitry (Mongiat & Schinder, 2011). These cells are therefore likely to be key contributors to the maintenance of cerebral function with ageing or following minor forms of trauma.

The effects of Ro61-8048 seen here on NeuN-containing cells may involve modulation of NMDA receptors. The direct administration of NMDA receptor-blocking agents prenatally or in the early postnatal period increases cell proliferation and overall granule neuron density in the dentate gyrus (Nacher *et al*., 2001; Maekawa *et al*., 2009), consistent with the increase in the number of NeuN-immunoreactive neurons in the hippocampal CA1 region and the dentate gyrus seen here. An overall increase in cell numbers is also reminiscent of the increased expression of proliferating cell nuclear antigen at P21 (Forrest *et al*., 2013a). On the other hand, several groups have reported that the administration of NMDA antagonists produces a loss of neurons and synapses in the CNS (Ikonomidou *et al*., 1999; Harris *et al*., 2003). Some of these variations may depend on the timing of drug administration and their effects on NMDA receptor subunit composition. In particular, the GluN2B subunit has been found to be especially important in cerebral development (Wang *et al*., 2011a), and, although expression of this subunit was elevated by Ro61-8048 in P21 rats, levels had normalised by P60 (Forrest *et al*., 2013a,b). The early change in its expression may nevertheless have permanently altered the sequence of events leading to new neuron formation.

### Doublecortin

Doublecortin is a microtubule-associated protein that is known to have key roles in early neuronal migration, especially of inhibitory interneurons (Friocourt *et al*., 2007; Cai *et al*., 2009), and is associated with recently generated neurons, for which it is frequently used as a diagnostic marker (Couillard-Despres *et al*., 2005; Jin *et al*., 2010). The highest concentrations of DCX correlate with sites of neurogenesis such as the subgranular zone of the hippocampal dentate gyrus (Francis *et al*., 1999), where it is expressed in post-mitotic cells in the adult during the initial phases of migration, when rapid dendritic growth is occurring (Spampanato *et al*., 2012). It is required for correct hippocampal lamination (Corbo *et al*., 2002), and its deletion results in reduced neurogenesis and poor recovery after stroke injury (Jin *et al*., 2010). The increase in hippocampal DCX levels therefore probably reflects increased neurogenesis following treatment with Ro61-8048 *in utero*. In fact, the direct observation of immunostained cells in this study indicates no change in the number of DCX-positive cells in the dentate gyrus and a decrease in the number of neurons bearing complex dendrites. This implies that the increase in protein expression reflects an increase in the numbers of recently generated neurons in other regions of the hippocampus. However, as no DCX-immunopositive cells were found in the CA1 and CA3 pyramidal layers, other positive cells may be primarily interneurons not studied here. In view of the substantial and ubiquitous increase in Shh levels across all hippocampal subfields, with Shh reflecting early cell maturation and tissue localisation, it may also be that new interneurons are migrating from the subgranular zone more rapidly after Ro61-8048 treatment, or at an earlier phase of the developmental sequence. Certainly, the developmental roles attributed to DCX temporally precede those ascribed to Shh, supporting the hypothesis that there is increased production of new neurons that then mature and differentiate more slowly than in control animals. This interpretation would be consistent with the normal number of granule cells overall but with fewer bearing extensive, mature, complex dendritic trees.

Functionally, the reduced numbers of dentate gyrus neurons with well-developed dendritic trees may imply increased excitability. Mutations of the DCX molecule can result in reduced synaptic inhibition (Kerjan *et al*., 2009), which is consistent with the neuronal co-localisation of DCX and GABA, together with accepted markers of GABA neurons such as parvalbumin (Cai *et al*., 2009) and evidence that many DCX-expressing neurons are destined to become GABAergic interneurons (Xiong *et al*., 2008). An increased excitability resulting from fewer DCX-containing inhibitory neurons could contribute to the reduced paired-pulse inhibition reported previously. There may also be another link to the early increased expression of GluN2B after Ro61-8048 treatment, as this subunit is important in the modulation of inhibitory interneuron activity (Hanson *et al*., 2013), and blockade of NMDA receptors by ketamine affects the hippocampal location of inhibitory, parvalbumin-containing interneurons (Sabbagh *et al*., 2013).

### Shh

The glycoprotein Shh and the functionally related group of Wingless proteins are involved in regulation of the cell cycle (Alvarez-Medina *et al*., 2009), as well as the early development of tissue polarisation and the generation of morphogenetic gradients (Jessell, 2000; Palma *et al*., 2005; Traiffort *et al*., 2010), cell proliferation, and the migration of progenitors to their functional destinations (Traiffort *et al*., 2001; Charytoniuk *et al*., 2002; Palma *et al*., 2005; Balordi & Fishell, 2007).

Despite its importance in the earliest stages of CNS formation, Shh continues to exist in the adult brain, notably in regions such as the cerebellum and hippocampus (Traiffort *et al*., 1998; Charytoniuk *et al*., 2002), where neurogenesis continues in the adult. The production of Shh may contribute to increased neurogenesis and recovery after brain damage, as the loss of Shh activity worsens such damage.

Expression of Shh appears to be especially sensitive to interference with the kynurenine pathway, and is downregulated in neonatal and adult animals by Ro61-8048 (Forrest *et al*., 2013a,b). The present results show a significant increase in the number of Shh-positive neurons in all three areas of the hippocampus examined as compared with control tissue. In the dentate gyrus, there is an additional shift in Shh localisation, with animals exposed to Ro61-8048 showing more cell bodies staining for the protein, but an increased level of diffuse staining in the intercellular space. As Shh is known to be a secreted protein (Traiffort *et al*., 2001), it is likely that this intercellular material reflects an increase in the secretion or leakage of Shh from the more dense pool of neurons.

Shh also plays a role in attraction and repulsion between developing synaptic terminals and their target areas of contact on neuronal somata or dendrites (Angot *et al*., 2008; Hor & Tang, 2010). The changes in Shh presence and cellular distribution may therefore constitute a factor affecting the dendritic rearrangements and complexity described above. In addition, as Shh has been linked to the development of inhibitory neurons such as cerebellar granule cells (Spassky *et al*., 2008), the changes in Shh expression and Shh-positive cell numbers may reflect differences in inhibitory neuron numbers or connectivity that could be partly responsible for the observed decreased in paired-pulse inhibition and long-term potentiation (Forrest *et al*., 2013b).

### VGLUT and VGAT

VGLUT-1 and VGLUT-2 have been widely adopted as indicators of excitatory glutamatergic transmission, the density of the transporters providing a valuable reflection of quantal size and transmitter release. VGLUT-1 is substantially more important in the adult hippocampus and associated behaviours (Balschun *et al*., 2010). VGLUT-2 is the predominant transporter in subcortical regions such as the thalamus and midbrain (Moechars *et al*., 2006; Kubota *et al*., 2007), especially during embryonic and neonatal development, being replaced by VGLUT-1 in adulthood (Fremeau *et al*., 2004). One of the most striking effects of prenatal Ro61-8048 exposure was an increase in the number of synaptic terminals showing VGLUT staining, with no change in the number of VAMP-1-positive terminals, confirming that there was no overall change in the total number of synaptic contacts. Equally, the absence of any change in VGAT staining confirms the absence of any global gain or loss of synapses, and reinforces the concept that the change in VGLUT-positive terminals is a highly specific phenomenon. It may be that this is a consequence of the increased proportion of dendritic branches available for synaptic contact relative to the decreased number and length of basal dendrites. It is well recognised that different groups of afferent neurons project onto different regions of the dendritic surface (Gidon & Segev, 2012; Ferrante *et al*., 2013). Although no attempt was made here to define the anatomical origin of the VGLUT-positive terminals counted, it is quite possible that the increase noted was confined to only one or a small number of afferent sources.

It is also possible that the increased number of VGLUT-positive terminals contributes to the reduced paired-pulse inhibition at small (10-ms) interpulse intervals (Forrest *et al*., 2013b). Although paired-pulse inhibition and facilitation are primarily determined presynaptically (Zucker *et al*., 1991; Rosenmund & Stevens, 1996), the final level of paired-pulse interaction is partly influenced by the number of excitatory and inhibitory terminals on the neurons being recorded. The larger number of excitatory terminals defined here could therefore be responsible for the reduced paired-pulse inhibition. It has also been noted by others that the expression of VGLUT is a factor in the relative and overall efficacy of excitatory and inhibitory neurotransmission (Fremeau *et al*., 2004; Wojcik *et al*., 2004; Wilson *et al*., 2005).

### The kynurenine pathway

Overall, this study reinforces the argument that the kynurenine pathway is active in the embryonic brain during early development. Components of the kynurenine pathway are present in neurons and glia at this time (Beal *et al*., 1992; Walker *et al*., 1999; Guillemin *et al*., 2001, 2005; Schwarcz & Pellicciari, 2002), the glia influencing the activation of NMDA receptors, possibly via the balance between the concentration of quinolinic acid (agonist) and that of kynurenic acid (antagonist). Indeed, the morphological organisation of developing tissues, including the brain, may be primarily defined by a gradient of antagonists such as kynurenic acid (Gurdon & Bourillot, 2001). Interfering with the kynurenine pathway during development would then significantly affect neuronal morphology and function in the hippocampus, as reported previously.

Indeed, any immune challenge to the mother or neonate that results, directly or indirectly, in the activation of central glia or peripheral macrophages, or changes in the levels of cytokines or kynurenines in the fetal or neonatal CNS, would alter the balance of quinolinic acid and kynurenic acid concentrations, and could seriously perturb neural development and plasticity. This could, in turn, increase the risk of CNS disorders, and would be consistent with evidence that genetic abnormalities of the kynurenine pathway are linked to disorders such as schizophrenia (Sathyasaikumar *et al*., 2011; Holtze *et al*., 2012; Stone & Darlington, 2013). The blockade of NMDA receptors by kynurenic acid produces neurochemical and behavioural changes that have been likened to those seen in schizophrenia (Harris *et al*., 2003; du Bois & Huang, 2007), and a significant amount of clinical evidence has identified elevated levels of kynurenic acid in the brains of schizophrenic patients (Linderholm *et al*., 2012; Stone & Darlington, 2013).

The focus in this study has been on the modulation of glutamate receptors, especially those responding to NMDA, by the kynurenine pathway. There are other recently identified sites at which kynurenic acid may also act to affect neuronal development or function (Stone *et al*., 2013), and one of these, the α7-nicotinic cholinoceptor, has received significant attention in recent years. Initial reports indicated that kynurenic acid was a potent antagonist at these receptors, although the high potency could not be confirmed (Stone, 2007). Indeed, more recent work has failed to find any antagonistic action of kynurenic acid at α7 receptors (Mok *et al*., 2009; Dobelis *et al*., 2012), suggesting that the original observations may have been made under highly selective conditions that are not generally applicable. The balance of evidence at present would therefore suggest that NMDA receptors remain the major site of action of kynurenic acid in the CNS. This would be consistent with the fact that NMDA receptors play key roles in the development and contact formation of neuronal growth cones (Wang *et al*., 2011b), the sprouting, stabilisation and length of dendritic branches (Sin *et al*., 2002; Kwon & Sabatini, 2011), and the specification of spine profile and density (Ultanir *et al*., 2007; Kwon & Sabatini, 2011). The implication of this study is that all of these may be under regulatory control by the kynurenine pathway and subject to environmental influences via this pathway.

## Abbreviations

CNS: central nervous system
DCX: doublecortin
E: embryonic day
KMO: kynurenine-3-monoxygenase
NMDA: *N*-methyl-d-aspartate
P: postnatal day
PBS: phosphate-buffered saline
Ro61-8048: 3,4-dimethoxy-*N*-[4-(3-nitrophenyl)thiazol-2-yl]benzenesulphonamide
Shh: sonic hedgehog
VAMP-1: vesicle-associated membrane protein-1
VGAT: vesicular GABA transporter
VGLUT: vesicular glutamate transporter
